# The Neighboring Subunit Is Engaged to Stabilize the Substrate in the Active Site of Plant Arginases

**DOI:** 10.3389/fpls.2020.00987

**Published:** 2020-07-10

**Authors:** Bartosz Sekula

**Affiliations:** Synchrotron Radiation Research Section of Macromolecular Crystallography Laboratory, National Cancer Institute, Argonne, IL, United States

**Keywords:** polyamine biosynthesis, urea cycle, agmatinase, ureohydrolases, arginine amidinohydrolase

## Abstract

Arginine acts as a precursor of polyamines in plants in two known pathways, agmatine and ornithine routes. It is decarboxylated to agmatine by arginine decarboxylase, and then transformed to putrescine by the consecutive action of agmatine iminohydrolase and N-carbamoylputrescine amidohydrolase. Alternatively, it can be hydrolyzed to ornithine by arginase and then decarboxylated by ornithine decarboxylase to putrescine. Some plants lack a functional ornithine pathway, but all have one or two arginases that can have dual cellular localization, in mitochondria and plastids. It was recently shown that arginases from *Arabidopsis thaliana* and soybean act also as agmatinases, thus they can produce putrescine directly from agmatine. Therefore, arginase (together with arginine decarboxylase) can complement putrescine production in plastids, providing a third polyamine biosynthesis pathway in plants. Phylogenetic analysis suggests that arginases, highly conserved in the plant kingdom, create the only group of enzymes recognized in the family of ureohydrolases in plants. Arginases are metalloenzymes with binuclear manganese cluster in the active site. In this work, two arginases from *A. thaliana* and *Medicago truncatula* are structurally characterized and their binding properties are discussed. Crystal structures with bound ornithine show that plant hexameric arginases engage a long loop from the neighboring subunit to stabilize α-amino and carboxyl groups of the ligand. This unique ligand binding mode is unobserved in arginases from other domains of life. Structural analysis shows that substrate binding by residues from two neighboring subunits might also characterize some prokaryotic agmatinases. This feature of plant arginases is most likely the determinant of their ability to recognize not only arginine but also agmatine as their substrates, thus, to act as arginase and agmatinase.

## Introduction

Arginine has the highest nitrogen to carbon ratio of all proteinogenic amino acids which makes it an effective storage form for organic nitrogen in plants; it may be responsible for up to half of the stored nitrogen in plant seeds (Winter et al., 2015). Therefore, arginine plays a critical role in nitrogen metabolism and recycling in plants (Slocum, 2005). Plants, unlike animals, rather recycle nitrogen in the form of urea instead of excreting it (Sirko and Brodzik, 2000). Except for being a building block for proteins, arginine (or its derivatives) is a potential source of nitric oxide (Flores et al., 2008). Moreover, under high nitrogen supply, arginine may secure proline production through degradation to ornithine (Forlani et al., 2015). Arginine can also be converted to putrescine, γ-aminobutyric acid, or nicotine, playing a key role in development and stress management in plants (Siddappa et al., 2018). Therefore, arginine catabolism is used not only to mobilize nitrogen reserves but also it is used as a part of plant defensive mechanism (Siddappa et al., 2018).

As an essential part of polyamine biosynthesis in plants, arginine can be used as a precursor of putrescine in several ways (Michael, 2016; Patel et al., 2017). In the first route, arginine undergoes decarboxylation by arginine decarboxylase to agmatine, which is then transformed to putrescine in two steps. The first reaction is catalyzed by agmatine iminohydrolase, the enzyme built of two subunits characterized by an αββαβ five-bladed propeller fold (Sekula and Dauter, 2019). The second step is carried out by N-carbamoylputrescine amidohydrolase, which in plants forms characteristic octamers with four pairs of subunits arranged helically (Sekula et al., 2016). An alternative route for putrescine production is the ornithine pathway. In this route, ornithine is obtained from arginine by the action of arginase (arginine amidinohydrolase, ARGAH). Then, putrescine is produced by ornithine decarboxylase. However, ornithine decarboxylase is missing in some plants (Hanfrey et al., 2001), therefore, a functional ornithine pathway is also absent in these species. The studies on ARGAH from *Arabidopsis thaliana* and soybean have also shown that concerted action of ARGAH and one of the arginine decarboxylases may complement the putrescine biosynthesis as the third route (Patel et al., 2017). Therefore, arginine is initially decarboxylated to agmatine and then, through the hydrolytic action of ARGAH on agmatine (agmatinase activity), putrescine is produced. These findings overturn earlier results (Chen et al., 2004) which indicated that tomato ARGAH (evolutionary closer related to *A. thaliana* ARGAH than soybean ARGAH), does not convert agmatine at high substrate concentration. ARGAHs in the plant kingdom are highly conserved and it was suggested that dual arginase and agmatinase activity of ARGAHs is common in plants (Patel et al., 2017). Moreover, ARGAH has high *Km* for arginine (Patel et al., 2017).Therefore, in plant tissues (or some cell compartments, like plastids), with low arginine concentration, arginine would be preferentially converted to agmatine by arginine decarboxylase, thus enabling the third putrescine biosynthesis pathway through agmatinase activity of ARGAH.

*A. thaliana* has two ARGAH isoforms encoded by genes *ARGAH1* (At4g08900) and *ARGAH2* (At4g08870), which most likely appeared by gene duplication (Brownfield et al., 2008). *Arabidopsis* ARGAHs present dual localization; they are localized in the mitochondrial matrix (Flores et al., 2008), but they can also be targeted to plastids (Patel et al., 2017). Only *ARGAH1* is actively transcribed in pollen (Brownfield et al., 2008), which likely secures the high demand of proline in pollen. In stress conditions (drought, oxidative stress, wounding) and upon methyl jasmonate treatment, *ARGAH2* expression is increased in a concerted manner with arginine decarboxylase 2 (Patel et al., 2017). Similarly, expression and activity of only one ARGAH isoform are alleviated as a response to wounding and methyl jasmonate in tomato leaves (Chen et al., 2004). ARGAH activity during germination is highly increased, which clearly shows that in plant seedlings arginine is used to recover nitrogen and carbon (Tiburcio et al., 1990). Thus, in germinating seeds (where most of the nitrogen is in the form of arginine), increased arginase activity (increased conversion of arginine to ornithine) is involved in the recovery of nitrogen and carbon from arginine to translocate it to growing points.

Similarly to other eukaryotic (Di Costanzo et al., 2010) and prokaryotic (Bewley et al., 1999) orthologues, plant ARGAHs, are binuclear manganese metalloenzymes. Together with agmatinase, formiminoglutamase, and proclavaminate aminohydrolase, ARGAHs are grouped with the Ureohydrolase Superfamily. This family of enzymes has a highly conserved fold (Dowling et al., 2008) with α/β/α sandwich. It also shares the mechanism of guanidine moiety hydrolysis (Christianson and Cox, 1999). However, it was suggested that plant arginases are unique in that they are phylogenetically more similar to the bacterial agmatinases, than to bacterial or mammalian arginases (Patel et al., 2017). A few ARGAHs have been described in plants (Boutin, 1982; Desai, 1983; Martin-Falquina and Legaz, 1984; Kang and Cho, 1990; Hwang et al., 2001; Dabir et al., 2005), however, the structure of any plant ureohydrolase is still unknown.

In this work, ARGAHs from two model plant species, *A. thaliana* (*At*ARGAH1) and *Medicago truncatula* (*Mt*ARGAH), are structurally characterized by X-ray crystallography and small-angle X-ray scattering (SAXS). Based on the crystal structures of *At*ARGAH1 and *Mt*ARGAH complexes with ornithine combined with the analysis of sequence conservation of the key residues of plant ARGAHs their dual arginase/agmatinase function in plants is discussed.

## Materials and Methods

### Cloning, Overexpression, and Purification of *Mt*ARGAH and *At*ARGAH1

Complementary DNA (cDNA) of *M. truncatula* and *A. thaliana* was obtained with the use of SuperScript II reverse transcriptase (Life Technologies), as well as oligo dT (15 and 18) primers and total RNA isolated from leaves with an RNeasy Plant Mini Kit (Qiagen). The open reading frames (ORF) of *At*ARGAH1 (Ordered Locus Name: At4g08900) and *Mt*ARGAH (Ordered Locus Name: MTR_4g024960) were isolated by polymerase chain reaction. Primers were designed to clone full ORF of *Mt*ARGAH and *At*ARGAH1 starting from codon 25. The following primers were used: TACTTCCAATCCAATGCCTCTGCTTCTTCAATCGAGAAAGGGCAAA (*At*ARGAH1-forward), TTATCCACTTCCAATGTTATCATTTCGAGATTTTCGCAGCTAATTCTCTAA (*At*ARGAH1-reverse), TACTTCCAATCCAATGCCATGTCGACTATAGCACGCAGAGG (*Mt*ARGAH-forward), TTATCCACTTCCAATGTTATCATTTTGACATCTTTGCAGCCAATTCTCT (*Mt*ARGAH-reverse).

A ligase-independent cloning (Kim et al., 2011) protocol was applied to incorporate *Mt*ARGAH and *At*ARGAH1 genes into a pMCSG68 vector (Midwest Center for Structural Genomics). The vectors with *At*ARGAH1 and *Mt*ARGAH were used to transform BL21 Gold *Escherichia coli* competent cells (Agilent Technologies). The correctness of the cloned sequences was checked by DNA sequencing of the isolated plasmids from the overnight culture in LB medium with 150 μg/ml of ampicillin. The pMCSG68 vector is used to express the construct with an N-terminal His_6_-tag followed by the tobacco etch virus (TEV) protease cleavage site.

Overexpression of the protein started with inoculation of 1 L of fresh lysogeny broth medium (with 150 μg/ml of ampicillin) with 15 ml of overnight culture. Then the medium was shaken at 37°C until OD_600_ reached value 1.0. Afterward, the culture was cooled to 10°C for 1 h and 0.5 mM of isopropyl-β-D-thiogalactopyranoside was added. After 16 h of overexpression at 18°C, the culture was cooled to 4°C. The cells were pelleted in the centrifuge (3,500×*g* for 20 min). The cell pellets were resuspended in 35 ml of the binding buffer (50 mM HEPES pH 7.8; 500 mM NaCl; 20 mM imidazole; 1 mM tris(2-carboxyethyl)phosphine, TCEP) and frozen at −80°C. Thawed samples were placed in an ice/water bath and subjected to sonication (60 four-second sonication bursts in 26-second intervals). Cell debris was pelleted in a centrifuge (25,000×*g* for 30 min at 4°C). The first step of protein purification was performed on HisTrap HP resin (GE Healthcare). The supernatant was transferred to the columns packed with 5 ml of resin which were coupled to Vac-Man (Promega). Then, the resin with bound *At*ARGAH1 and *Mt*ARGAH was washed five times with 40 ml of the binding buffer. *At*ARGAH1 or *Mt*ARGAH were eluted with 20 ml of elution buffer (50 mM HEPES pH 7.8, 500 mM NaCl, 400 mM imidazole, 1 mM TCEP). Cleavage of the His_6_-tag from *At*ARGAH1 and *Mt*ARGAH by His_6_-tagged TEV protease (final concentration of 0.1 mg/ml) was performed in parallel to overnight dialysis at 4°C against the buffer: 50 mM HEPES pH 7.8, 500 mM NaCl, 1 mM TCEP. Cleaved His_6_-tag and His_6_-tagged TEV protease were separated from *At*ARGAH1 and *Mt*ARGAH on HisTrap HP resin. Size-exclusion chromatography on a HiLoad Superdex 200 16/60 column (GE Healthcare) coupled to the AKTA FPLC system (Amersham Biosciences) was the last step of purification. The running buffer was as follows: 50 mM HEPES pH 7.8, 100 mM KCl, 50 mM NaCl, 1 mM TCEP.

### Crystallization and Data Collection

*At*ARGAH1 and *Mt*ARGAH were concentrated with Amicon concentrators (Millipore) to the final concentration of approximately 18 and 6 mg/ml, respectively. Concentration was determined by the absorbance measurement at 280 nm with the following extinction coefficients: 18,910 M^−1^ × cm^−1^ (*Mt*ARGAH) and 14,440 M^−1^ × cm^−1^ (*At*ARGAH1). Proteins were crystallized by the hanging drop method; the best crystals were obtained with streak seeding. *Mt*ARGAH was crystallized in conditions optimized from initial screening in Morpheus Screen (Molecular Dimensions): 55 mM CaCl_2_, 55 mM MgCl_2_, 80 mM HEPES/MOPS buffer at pH 7.5, 30% ethylene glycol; 15% polyethylene glycol 8000. *At*ARGAH1-ORN was crystallized in conditions optimized from initial screening in BCS screen (Molecular Dimensions): 50 mM L-arginine, 50 mM L-glutamic acid, 22% PEG Smear Broad, 5% glycerol. The complex of *Mt*ARGAH-ORN was obtained by cocrystallization with 50 mM L-arginine. Crystal of *At*ARGAH1-ORN was cryoprotected with glycerol.

The diffraction data were collected at the SER-CAT 22-ID and SBC 19-ID beamlines at the Advanced Photon Source (APS), Argonne National Laboratory, USA. The diffraction data were processed with *XDS* (Kabsch, 2010) and *HKL-3000* (Otwinowski and Minor, 1997); for details see **Table 1**.

**Table 1:** Data collection and refinement statistics.

Structure:	*Mt*ARGAH	*Mt*ARGAH-ORN	*At*ARGAH1-ORN
**Data collection**			
Beamline	19-ID	22-ID	22-ID
Wavelength (Å)	0.979	1.00	1.00
Temperature (K)	100	100	100
Space group	*P*2_1_	*P*2_1_	*I*4_1_
Unit cell parameters *a b c* (Å) *β* (°)	79.3 142.9 90.0 115.9	83.7 166.1 150.9 94.5	267.3 267.3 262.9 90.0
Oscillation range (°)	0.3	0.5	0.25
Resolution (Å)	44.55–1.93 (2.04–1.93)	50–2.12 (2.25–2.12)	30–2.25 (2.33–2.25)
Reflections collected/unique	457,132/130,001	971,248/231,608	1,434,604/429,624
Completeness (%)	95.7 (93.6)	99.7 (98.9)	99.0 (99.9)
Multiplicity	3.5 (3.4)	4.2 (4.0)	3.3 (3.4)
*R*_merge_ (%)	7.5 (62.6)	5.6 (71.4)	6.4 (48.3)
<*I*/σ(*I)*>	10.9 (2.1)	15.5 (1.9)	17.1 (2.5)
**Refinement**			
*R*_free_ reflections	1,040	1,019	2,236
No. of atoms (non-H)			
protein	14,733	29,581	58,189
ligands	12	87	368
solvent	547	1,331	4,951
*R*_work_/*R*_free_ (%)	18.2/21.8	16.0/19.9	15.9/19.7
Mean ADP^a^ (Å^2^)	36.0	51.0	41.3
RMSD from ideal geometry			
bond lengths (Å)	0.01	0.01	0.01
bond angles (°)	1.9	0.9	1.8
Ramachandran statistics (%)			
favored	96	98	97
allowed	4	2	3
outliers	0	0	0
PDB code	6VSS	6VST	6VSU

^a^ADP, atomic displacement parameter.

Values in parentheses refer to the highest-resolution shell.

### Structure Determination and Refinement

The structure of unliganded *Mt*ARGAH was solved in *Phaser* (McCoy et al., 2007). The structure of putative agmatinase from *C. difficile* (PDB ID: 3LHL) was used as the search model. The initial solution was rebuilt in *PHENIX AutoBuild* (Terwilliger et al., 2008). Then, the structure underwent the subsequent steps of manual and automatic refinement with *Coot* (Emsley et al., 2010) and *REFMAC* (Murshudov et al., 2011). Unliganded *Mt*ARGAH was used as the search model for the determination of *Mt*ARGAH-ORN and *At*ARGAH1-ORN structures. TLS parameters (Winn et al., 2001; Winn et al., 2003) were used in the later stages of the structure refinement. Standard CCP4 libraries (Winn et al., 2011) were used for the refinement of ligands in *REFMAC* or *PHENNIX* (Adams et al., 2010). Polder omit maps (Liebschner et al., 2017) were used to validate the position of bound ornithine in *Mt*ARGAH-ORN and *At*ARGAH1-ORN (**Figures 5A, B** **)**. The quality of refined structures was controlled by *R*_work_, *R*_free_ (Brunger, 1992) and geometric parameters. Evaluation of the final models was done in *MolProbity* (Chen et al., 2010). CheckMyMetal (Zheng et al., 2017) was used for the evaluation of the geometry of bound Mn^2+^ ions. The final refinement statistics are given in **Table 1**.

### Small-Angle X-Ray Scattering (SAXS) Measurements

SAXS data were collected from *At*ARGAH1 (5.5 mg/ml) and *Mt*ARGAH (5 mg/ml) at the BioCAT 18-ID beamline (Fischetti et al., 2004) at APS with the in-line size exclusion chromatography setup and Pilatus3 1M detector (Dectris). Prior to the SAXS data collection, the sample was applied to the WTC-015S5 column (Wyatt Technologies) coupled to the Infinity II HPLC (Agilent Technologies) system. Directly after the separation on the column samples were analyzed with the Agilent UV detector, a Multi-Angle Light Scattering (MALS) detector and a Dynamic Light Scattering (DLS) detector (DAWN Helios II, Wyatt Technologies), and a RI detector (Optilab T-rEX, Wyatt), and then the samples were directed to the SAXS flow cell (1.5 mm quartz capillary). The scattering data were collected at 1.03 Å wavelength at room temperature with 0.5 s exposure every 2 s. The sample-to-detector distance was 3.5 m and the collected q-range was 0.004–0.4 Å^−1^. Data reduction and analysis were performed in *BioXTAS RAW* 1.5.1 (Hopkins et al., 2017). Several frames from the elution peak and were averaged, which increased the signal-to-noise ratio. Then, the buffer signal from averaged frames proximal to the sample peak was subtracted from the averaged scattering data of the elution peak. The *Rg* values calculated from the Guinier and distance distribution analysis were 36 Å for both analyzed proteins. The calculated maximum dimensions of the particles (*D*_max_) for *At*ARGAH1 and *Mt*ARGAH were almost identical, 104 and 105 Å, respectively. Further calculations were done with the following *qRg* limits: 0.45–1.31 (*At*ARGAH1) and 0.36–1.31 (*Mt*ARGAH). *DAMMIF* (Franke and Svergun, 2009), *DAMAVER* (Volkov and Svergun, 2003), *DAMMIN* (Svergun, 1999) and *DAMFILT* were consecutively used for the calculation of the *ab initio* envelopes, averaging, refinement and filtration. Threefold symmetry restraints were applied during the envelope generation. SAXS envelopes were superposed with the crystallographic hexamers of *At*ARGAH1 and *Mt*ARGAH in *SUPCOMB*.

### Phylogenetic Analysis

The first set of sequences of *Viridiplantae* protein sequences, annotated as a Superfamily of ureohydrolases (IPR006035) in the *InterPro* database (Finn et al., 2017), was filtered by *ElimDupes* (www.hiv.lanl.gov) to obtain a set of unique sequences. Then, sequences were sorted in *BioEdit* (Hall, 1999); sequences with the length of 300–430 residues were used for further analysis. This set was aligned by *MUSCLE* (Edgar, 2004) in *MEGA7* (Kumar et al., 2016). Clear outliers and incomplete sequences were removed manually. The final set contained 141 sequences, which were re-aligned in *MUSCLE* and conservation of each residue was analyzed.

The second set was created by the search of non-redundant protein sequences database in protein *BLAST* (Altschul et al., 1997) with the sequence of *At*ARGAH1 without predicted signal peptide (UNIPROT ID: P46637). Results with E value lower than 0.005 were further analyzed analogically to the Set 1. Final set consisted of 226 sequences (the full list of the accession numbers of analyzed sequences can be found in the **Supplementary Material**).

### Other Software Used

Molecular illustrations were created with UCSF *Chimera* (Pettersen et al., 2004) and *PyMOL* (Schrödinger, LLC). The secondary structure was recognized with ProMotif (Hutchinson and Thornton, 1996) within the *PDBsum* server (de Beer et al., 2014). Sequence alignments were edited in *BioEdit* (Hall, 1999).

## Results and Discussion

### The Structure of *At*ARGAH1 and *Mt*ARGAH

In *A. thaliana*, there are two ARGAHs (*At*ARGAH1 and *At*ARGAH2), while *M. truncatula* has only one isoform, *Mt*ARGAH. The subunits of these plant ARGAHs have 342 (*At*ARGAH1), 344 (*At*ARGAH2), 338 (*Mt*ARGAH) residues. *At*ARGAH1 and *At*ARGAH2 share almost 85% identity. *Mt*ARGAH presents 76 and 72% sequence identity to *At*ARGAH1 and *At*ARGAH2, respectively. They all have signal peptide (21-residue long in *At*ARGAH1, 26 residues in *At*ARGAH2, and 15 residues in *Mt*ARGAH) (Almagro Armenteros et al., 2019) that is predicted to target proteins to mitochondria. However, the studies show that *Arabidopsis* ARGAHs present dual localization, they can also be targeted to plastids (Patel et al., 2017). In fact, about 20 N-terminal residues are disordered in the structures of full-length *Mt*ARGAH and they were not modeled in the final models.

**Figure 1:**
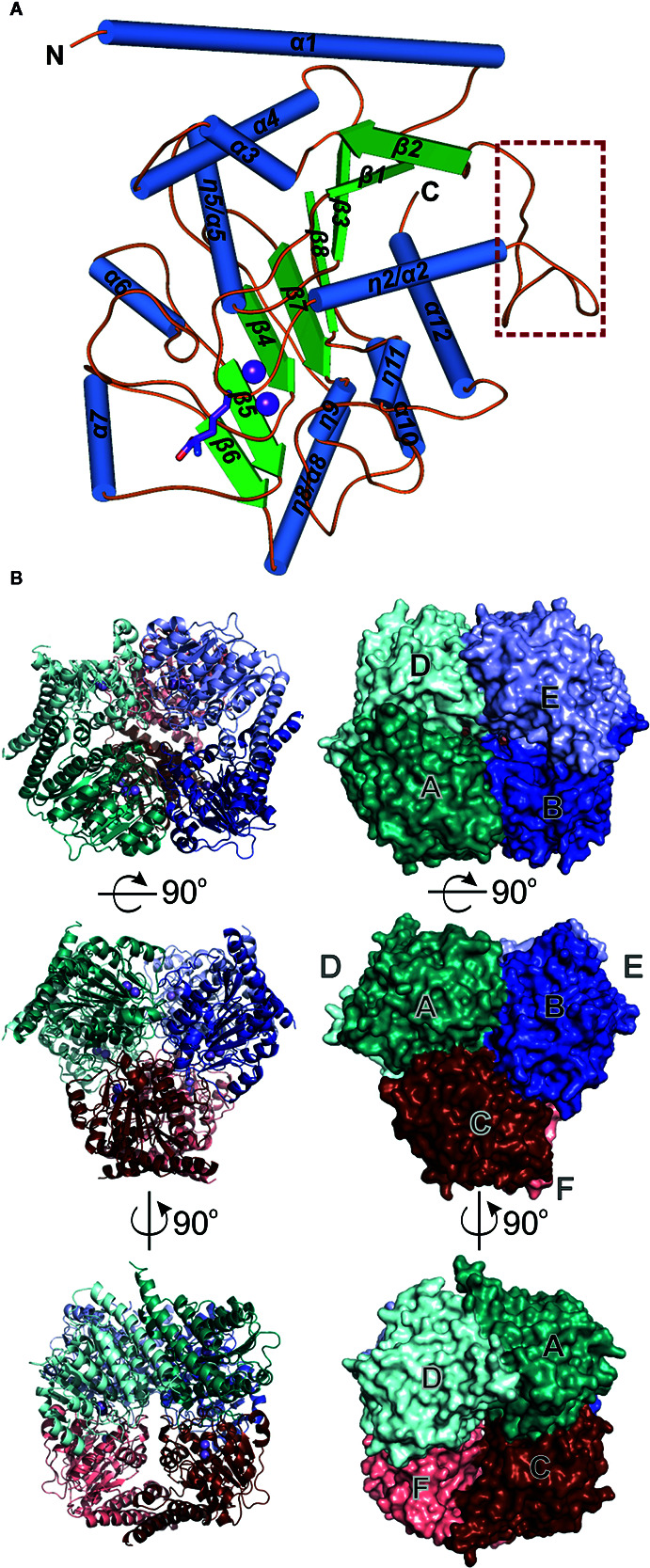
ARGAH crystal structure. **(A)** Architecture of *At*ARGAH1 monomer, secondary structure elements are color-coded as follows: helices (blue cylinders), sheets (green arrows), and coil regions (orange lines); the loop L2* (engaged in substrate binding in the neighboring subunit) is marked with a brown square. **(B)** *At*ARGAH1 hexameric assembly shown in cartoon (left) and surface (right) representation; each subunit is depicted with different color.

*At*ARGAH1 and *Mt*ARGAH share the arginase/deacetylase fold (Andreeva et al., 2008). The subunit architecture in both proteins is the three-layer α/β/α sandwich where the eight-stranded parallel β-sheet is buried between two helical bundles (**Figure 1A**). Five helices cover one face of the β-sheet and the set of six helices is placed on the other side. Structures of both ARGAHs are very similar, with about 0.7 Å RMSD of the superposed Cα atoms of corresponding monomers. Both proteins have two *cis*-peptide bonds (Glu150-Pro1151 and Gly158-Gly159 in *At*ARGAH1; Asp146-Pro147 and Gly154-Gly155 in *Mt*ARGAH). The *cis*-peptide bond between two Gly residues is highly conserved in ureohydrolases, it is in the conserved Gly-Gly-Asp-His motif (Ahn et al., 2004), which contributes to the region responsible for manganese binding within ARGAH active site (see below). A characteristic feature of plant ARGAHs is the protruding loop region (L2*, **Figure 1A**), which shapes the active site entrance of the neighboring subunit within the oligomer (see below).

**Figure 2:**
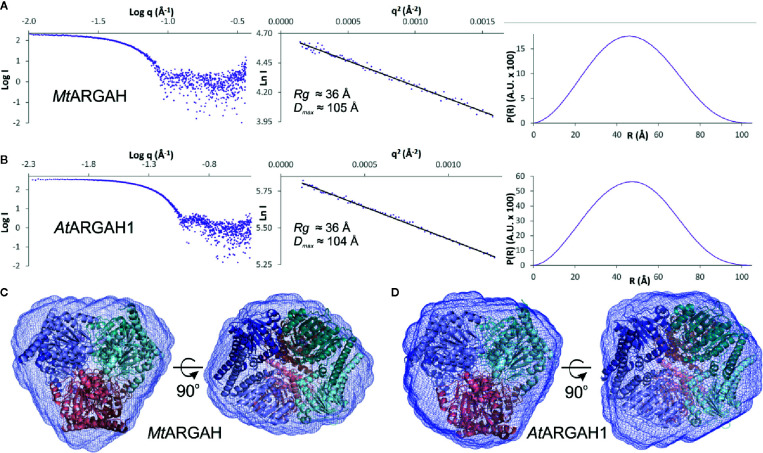
ARGAH solution structure. SAXS data for *Mt*ARGAH **(A)** and *At*ARGAH1 **(B)**; left chart presents the experimental SAXS curve; Guinier plots of the scattering curve with the best fit shown as a black line are shown in the center; pair-distance distribution function for SAXS data, P(R), is shown on the right. *Ab initio* averaged SAXS envelopes (blue mesh) of *Mt*ARGAH **(C)** and *At*ARGAH1 **(D)**; envelopes are superposed with *Mt*ARGAH and *At*ARGAH1 crystallographic hexamers.

The crystal structures of both ARGAH enzymes indicate that they also share the same symmetrical hexameric assembly (32 symmetry, **Figure 1B**); RMSD of the Cα atoms of superposed hexamers of *At*ARGAH1-ORN and *Mt*ARGAH-ORN is ~1 Å. The analysis with PISA server (Krissinel, 2015) shows that both ARGAHs present similar total buried area (~21,000 Å^2^). The hexamer is formed by a pair of three subunits (ABC and DEF, each with the threefold symmetry, center panel of **Figure 1B**) which are stacked with one another in a way that each subunit from one triplet (A, B, C) interacts with only one subunit from another triplet (D, E, F, respectively). In fact, the pair of triplets in *Mt*ARGAH seems to be tighter than it is in *At*ARGAH1; the buried area between interacting monomers A/D, B/E, C/F in *Mt*ARGAH is in average ~1,600 Å^2^, while in in *At*ARGAH1 it is ~1,200 Å^2^. SAXS results for both proteins (**Figures 2A, B** **)** are very similar, with identical *Rg* and nearly identical *D_max_*, and confirm that plant ARGAHs are also hexamers in solution. The estimated molecular weight of *At*ARGAH1 from SAXS data (211 kDa) matches almost ideally the hexameric *At*ARGAH1 (calculated molecular weight of the expressed hexameric construct is 209 kDa). Although the predicted molecular weight of *Mt*ARGAH from SAXS (191 kDa) differs from the theoretical hexamer mass based on the sequence of expressed *Mt*ARGAH (224 kDa), the calculated *ab initio* SAXS envelope of *Mt*ARGAH still represents the *Mt*ARGAH hexamer (**Figure 2C**). It is, in fact, more detailed than the SAXS envelope of *At*ARGAH1 (**Figure 2D**) and slightly more resembles the crystal structure.

### Active Site

**Figure 3:**
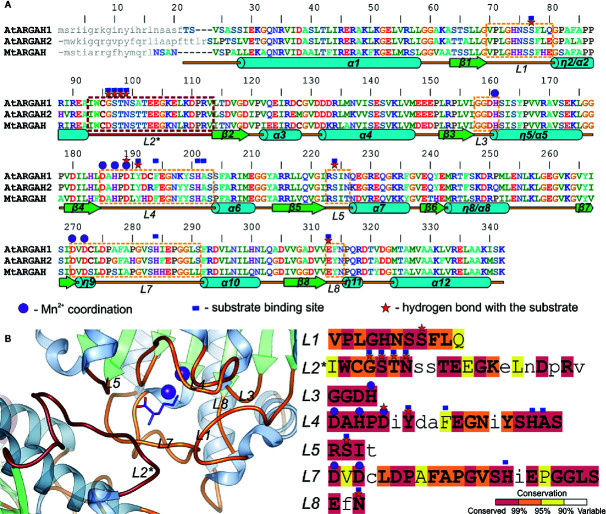
ARGAH structure. **(A)** Sequence alignment of *At*ARGAH1, *At*ARGAH2, and *Mt*ARGAH; residues are color-coded by type; secondary structure elements are shown below the alignment: helices (cylinders), sheets (arrows), and coil regions (lines); dashed lines mark sequence regions of the active site vicinity; loop L2* (engaged in substrate binding in the neighboring subunit) is marked in brown; purple circles denote residues engaged in coordination of the manganese cluster; red stars mark residues that are involved in the hydrogen bonding interactions with the substrate; blue squares indicate the residues that build the substrate binding site (in 5 Å radius of ornithine bound in ARGAH structures); grey small letters mark the predicted signal peptides; sequence positions above the alignment refer to *At*ARGAH1 sequence. **(B)** Architecture of the substrate binding site in *At*ARGAH1 structure (left) and sequence conservation of plant ureohydrolases (calculation is based on a set of set of 226 unique sequences, see *Materials and Methods*) in the loop regions that build the active site entrance; residues are highlighted accordingly to the presented conservation score; small letters depict less conserved residues.

**Figure 4:**
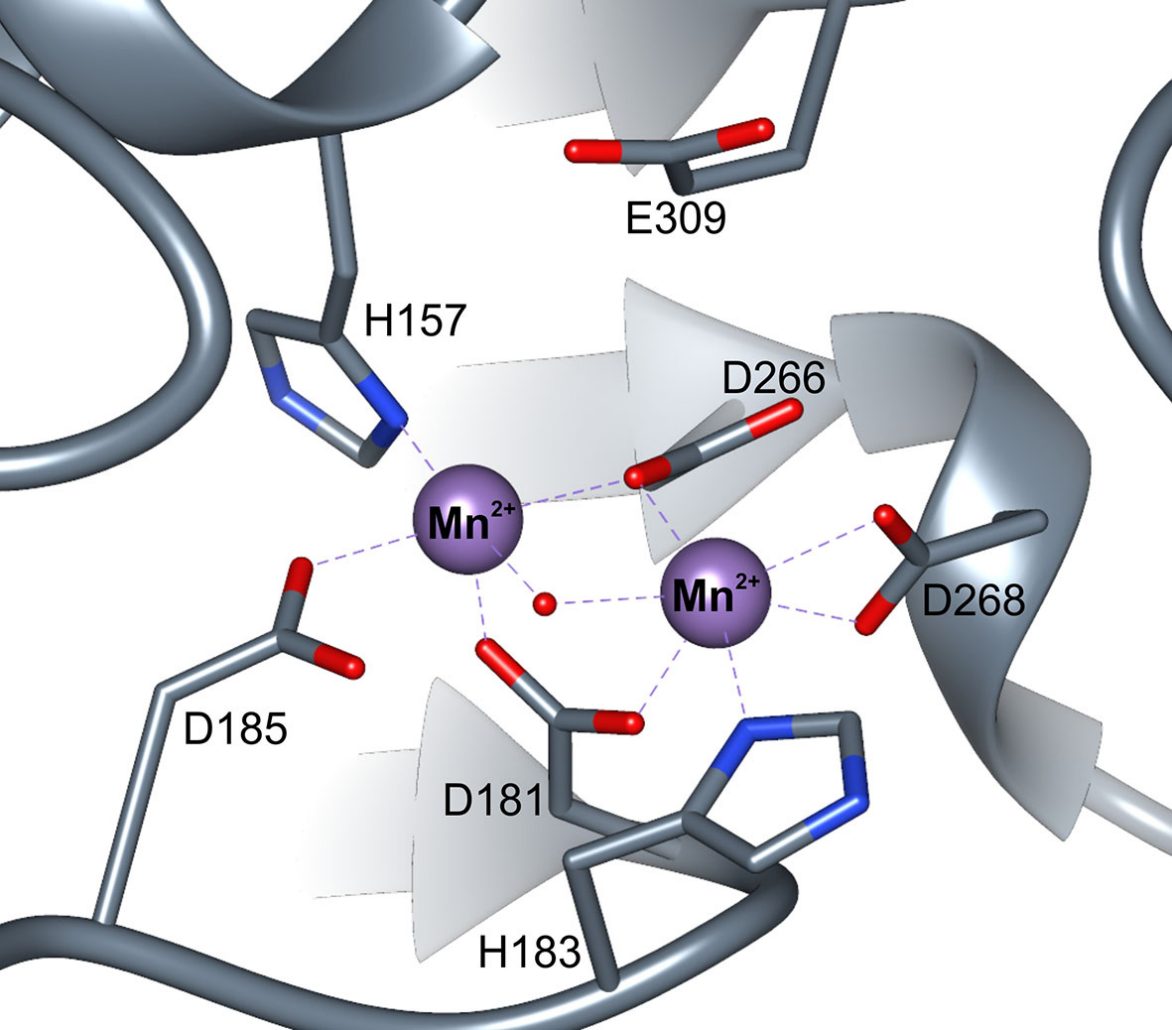
ARGAH active site. Coordination of manganese ions (purple spheres) in the active site of *Mt*ARGAH structure; red dot represents the catalytic hydroxide ion.

The active site with a double manganese cluster is formed in each ARGAH subunit by loop regions (*L1*, *L3*, *L4*, *L5*, *L7*, and *L8*) placed on the C-terminal ends of six β-stands (**Figure 3**). Additionally, the entrance of the active site is complemented by the loop L2* from the neighboring subunit in the triplet. The amino acid composition of these loops is very similar in described ARGAHs (**Figure 3A**). Moreover, based on the phylogenetic analysis (see *Materials and Methods* section) these loops exhibit highly conserved features in all plant ureohydrolases (**Figure 3B**, see below). Thus, the structural characteristics of the active site of *At*ARGAH1 and *Mt*ARGAH can be transferred with high probability to other plant ARGAHs, as well. The manganese cluster is placed deep inside the active site, close to the C-terminal ends of β4 and β7 on one face of the core β-sheet (**Figures 3B** and **4**). Two Mn^2+^ ions are bridged together in *Mt*ARGAH by Asp181, Asp266, and the catalytic hydroxide ion (**Figure 4**, see below). In *At*ARGAH1 the bridging residues are Asp185 and Asp270. The general coordination geometry of Mn^2+^ ions in ARGAH active site is square pyramidal and distorted octahedral (Christianson and Cox, 1999). In *Mt*ARGAH, Mn^2+^ ions present the same coordination geometry (**Figure 4**). Square pyramid around one Mn^2+^ is formed by OD2 of Asp185, ND1 of His157, OD2 of Asp266, OD1 of Asp181, and the bridging hydroxide ion (OD2 of Asp181 is perpendicular to the plane formed by the other four ligands). Octahedral coordination of the other Mn^2+^ is created by OD1 of Asp181, ND1 of His183, OD2 of Asp266, OD1 of Asp268, OD2 of Asp268, and the bridging hydroxide ion. The architecture of the region responsible for Mn^2+^ binding is the same in *At*ARGAH1 (**Figure 3A**), and it is highly conserved not only in plant ARGAHs (**Figure 3B**) but also in arginases from other domains of life (Christianson and Cox, 1999). In some structures of ARGAHs with ligands (Ahn et al., 2004), both Mn^2+^ have disordered octahedral coordination geometry with an additional water molecule, which is also observed in some subunits in *At*ARGAH1-ORN structure.

Plant arginases are likely not different from other manganese ureohydrolases regarding the catalytic mechanism, widely accepted for these enzymes (Christianson and Cox, 1999). Catalytic hydroxide ion (the one which coordinates both Mn^2+^), initially is H-bonded to Asp185 in *Mt*ARGAH structure. The substrate (arginine or agmatine) enters the active site with guanidine moiety placed above the manganese cluster. Glu309 in *Mt*ARGAH (Glu313 in *At*ARGAH1), localized at the back wall of the catalytic cavity, likely H-bonds the substrate so it can be oriented in a way that the center carbon atom of the guanidine moiety is in close vicinity to the catalytic hydroxide ion. Eventually, the hydroxide ion performs a nucleophilic attack on the central carbon of the substrate to create a tetrahedral intermediate, similar in geometry to ARGAH inhibitors (Di Costanzo et al., 2005). After a proton transfer, likely from Asp185, to the amino group of created ornithine (or putrescine), the intermediate is broken down with the release of reaction products: ornithine (putrescine) and urea.

### Plant ARGAHs Engage Second Subunit to Stabilize the Substrate in the Active Site

**Figure 5:**
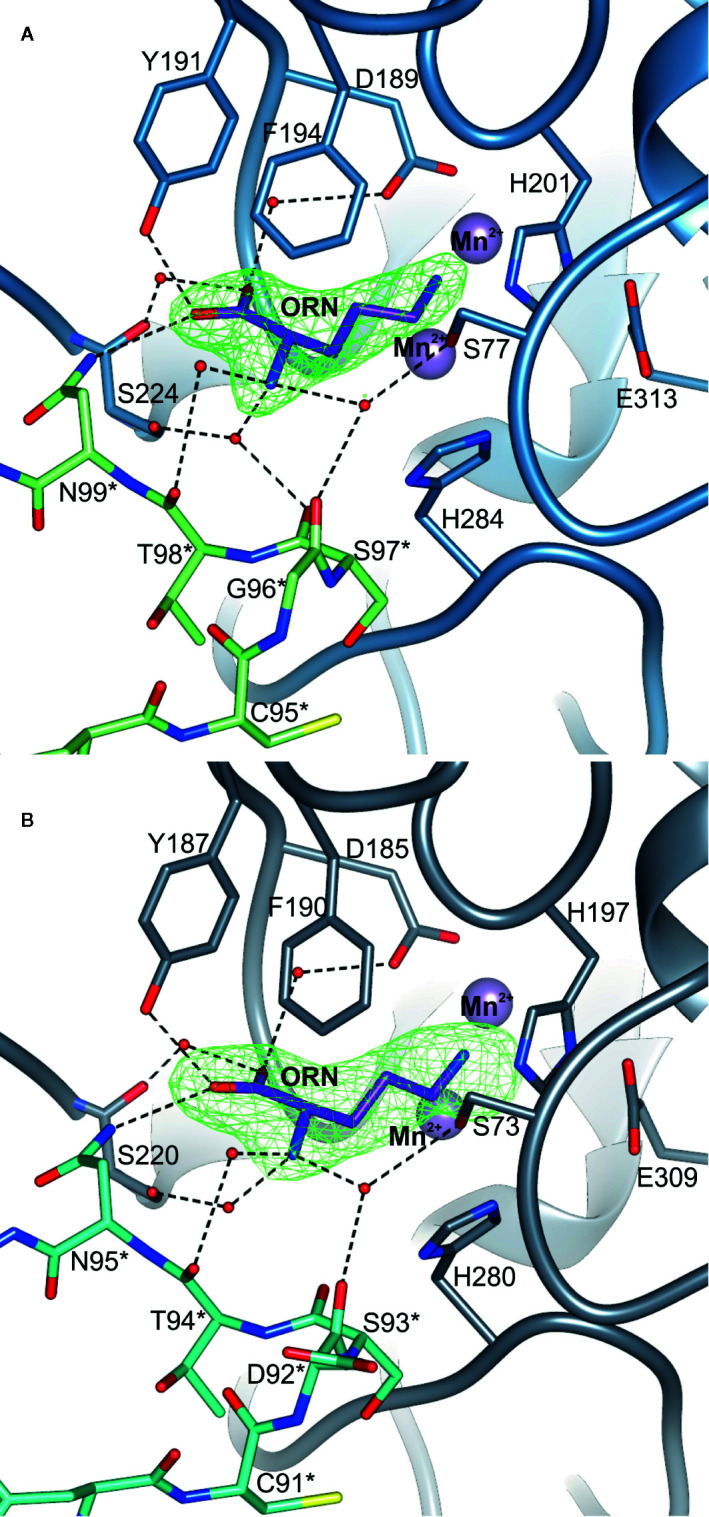
The ligand binding mode of plant ARGAHs. The binding mode of ornithine (ORN) in the active site of **(A)** *At*ARGAH1 and **(B)** *Mt*ARGAH; dashed lines indicate hydrogen bonds; residues are numbered accordingly to the sequence positions of presented proteins; green mesh represents Polder omit maps (contoured at 6 σ) around bound ornithine calculated in *Phenix* (Liebschner et al., 2017); residues from the neighboring subunit are marked with asterisk.

Structures of *At*ARGAH1-ORN and *Mt*ARGAH-ORN with the reaction product, ornithine, were obtained as a result of *in vitro* arginine hydrolysis. Although the hydrolysis took place before the crystal growth (there is no trace of urea molecule in the active site, and ornithine is not uniformly bound in all ARGAH active sites), the structures clearly correspond to the post-catalytic state of the enzyme. This inhomogeneity in ornithine binding can be explained by insufficient concentration of the ligand, since ARGAHs should present relatively low affinity to the reaction product. However, in subunits where electron density maps are more clear and indicate ornithine binding (**Figures 5A, B** **)**, the position of the ligand in the model explains the ligand binding mode of plant ARGAHs.

**Figure 6:**
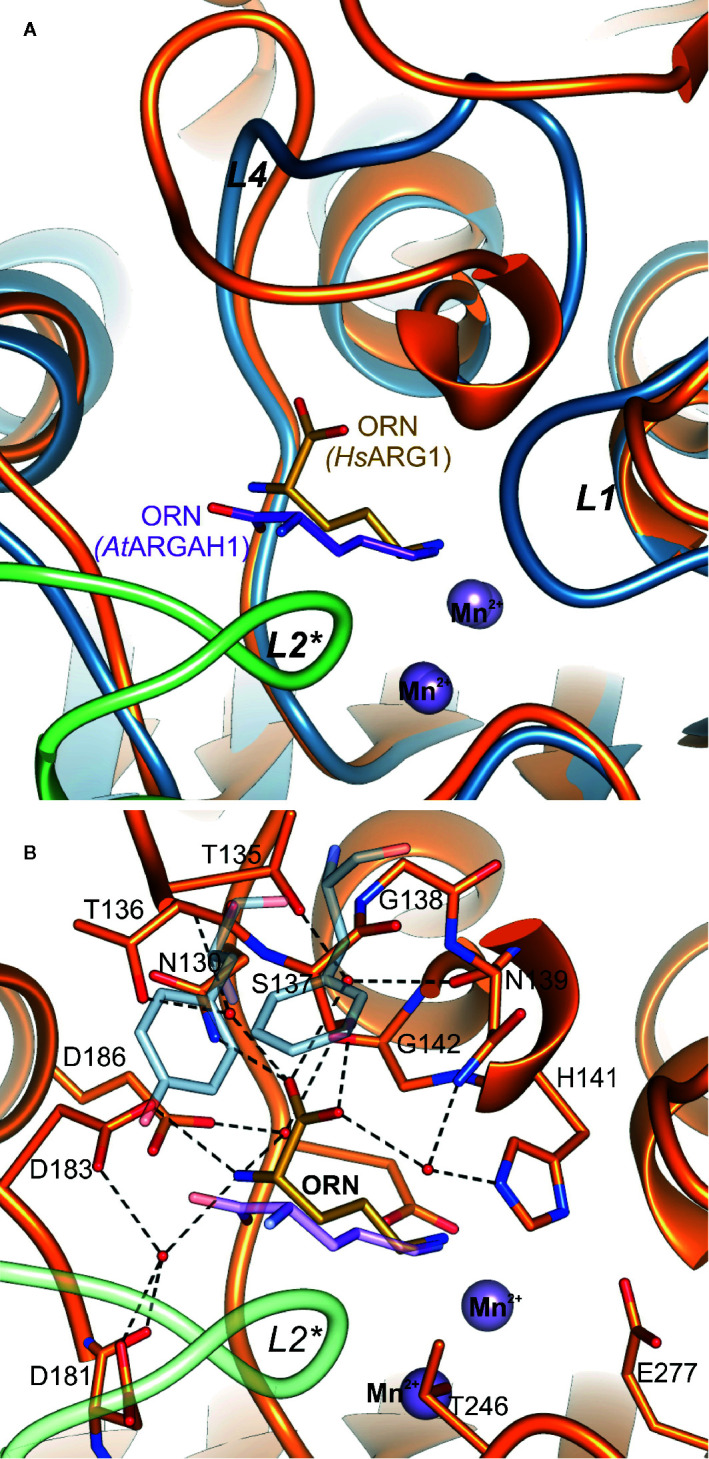
Comparison of ornithine binding mode in *At*ARGAH1 and *Hs*AGS1. **(A)** Cartoon representation of the superposed *At*ARGAH1-ORN and *Hs*ARG1-ORN (PDB ID: 3GMZ) structures; the chain of subunit A and B of *At*ARGAH1 is light blue and green, while the chain of *Hs*ARG1 is orange; **(B)** interactions of bound ornithine (yellow) in the structure of *Hs*ARG1-ORN (orange, PDB ID: 3GMZ); to highlight structural differences of plant ARGAHs *Hs*ARG1 some structural features (the loop L2*, ornithine, Phe191, and F194) of the superposed *At*ARGAH1 structure are shown in semi-transparent representation.

Ornithine penetrates the active site with Nϵ amino group pointed towards the manganese cluster. Therefore, α-amino and carboxyl groups of the ligand are stabilized by residues from *L*1, *L*4, *L*5, *L*7 coil regions of one subunit, and *L*2* from the neighboring subunit of ARGAH hexamer (**Figures 5A, B** **)**. At the active site entrance of *Mt*ARGAH, carboxyl group of ornithine creates two direct hydrogen bonds with the hydroxyl group of Tyr187 and the amino group of Asn95 from the neighboring subunit (Tyr191 and Asn95 in *At*ARGAH1). Additionally, it creates a couple of water-mediated H-bonds with Ser220, with Asp185 (Ser224, with Asp189 in *At*ARGAH1). The amino group of ornithine is more exposed to the solvent region. As a result, it is H-bonded to three water molecules that mediate the interactions with Ser73, Ser220, Asp92*, and Thr94* (Ser77, Ser224, Gly96*, Ser97*, and Thr98* in *At*ARGAH1; asterisk denotes residue from the neighboring subunit). In some monomers of *Mt*ARGAH, the interatomic distances also suggest direct H-bond interaction of an amino group of ornithine with Ser93*. Somewhat limited resolution of the structures and partial disorder in the solvent region does not allow of an unambiguous assignment of the interactions of α-amino group of ARGAH substrate with the main chain of L2* region (Asp92*, Ser93*, and Thr94* of *Mt*ARGAH; Gly96*, Ser97*, and Thr98* of *At*ARGAH1). The interactions could also vary depending on the conditions (pH or ionic strength). Therefore, when agmatine (lacking a carboxyl group) would be the bound ligand, this substrate would not interact with Tyr187 and Asn95 (Tyr191 and Asn99 in *At*ARGAH1). Thus, the amino group of agmatine could be moved closer to residues from L2*, where it could possibly create direct hydrogen bonds with their carbonyl oxygen atoms. Interestingly, orientation and interactions with the surrounding residues of the α-amino and carboxyl groups of bound ornithine in plant ARGAHs are nowhere near orientation of ornithine in human arginase (**Figure 6**) (Ilies et al., 2011) and other arginases (see below).

### Plant ARGAHs Present Highly Conserved Features Around the Active Site Entrance

*InterPro* database (Finn et al., 2017) contains only five sequences of *Viridiplantae* proteins annotated as arginases. Therefore, to investigate the sequence conservation of plant ARGAHs, a set of 226 unique sequences were used in the analysis (see *Materials and Methods*). This set contained nearly all sequences assigned to the superfamily of *Viridiplantae* ureohydrolases (IPR006035). It was used to analyze the conservation of residues from *L*1–*L*5, *L*7 and *L*8 regions that build the ARGAH active site and its entrance (**Figure 3B**). These are residues that are responsible for the substrate specificity of plant ureohydrolases.

It is no surprise that the sequence of plant ureohydrolases is fully conserved in the region responsible for the interactions with double manganese cluster since they hydrolyze guanidine moiety of the substrate. More unexpected is that almost all residues from the vicinity (5 Å radius) of bound ornithine in presented plant ARGAHs structures (*At*ARGAH1-ORN and *Mt*ARGAH-ORN) are highly conserved (conservation >99%, **Figure 3B**). Only two residues in *L*2*, corresponding to Gly96* and Thr98* of *At*ARGAH1, and one in *L*4 (Phe194 of *At*ARGAH1) present lower conservation (these positions are identical in >95% of analyzed sequences). However, the slight variability of Gly96-Ser97-Thr98 fragment concerns the region that interacts with ornithine *via* carbonyl oxygens of the main chain. In this case the overall conformation of the *L*2* loop is more important than the type of side chains of amino acids which build the loop.

The helix η2/α2 in the ARGAH structure is followed by a pair of hydrophobic residues (Ile or Met in position equivalent to residue 93 of *At*ARGAH1, and the fully conserved Trp94) which bends the main chain to begin the β-turn, just before the Ser97-Thr98-Asn99 motif. In that way, Asn99 and carbonyl oxygens of the preceding residues are positioned close to the entrance of the active site to bind the substrate. Comparison of *At*ARGAH1 with *Mt*ARGAH shows that substitution of glycine to aspartic acid (**Figures 5A, B** **)** does not affect the conformation of the loop *L*2*. Therefore, evolutionary pressure in this region is to preserve the overall conformation of the main chain rather than to conserve side chains. Additionally, the residue which H-bonds carboxyl group of ornithine (Asn99 in *At*ARGAH1 and Asn95 in *Mt*ARGAH) is fully conserved in all plant ARGAHs.

Another important region for ARGAH specificity is loop *L*4 with fully conserved Tyr191 that also binds carboxyl group of the substrate. In this case, two consecutive β-turns position Phe194 and Tyr191 close to each other and in the vicinity of the substrate. Phe194 from *L*4 in 16 sequences from the analyzed set is substituted with tyrosine. Therefore, this position requires an aromatic residue which limits the size of the active site. Interestingly, Tyr191 is flanked by more variable residues in plant ureohydrolases; positions 192 and 193 present especially high variability. However, in both, *At*ARGAH1 and *Mt*ARGAH, substitution of the side chains of residues corresponding to positions 192 and 193 has minor effect on the position of the main chain in this region and side chains of Phe194 and Tyr191.

Overall, the conservation of loop regions around the active site is very high in all plant ureohydrolases, not only in terms of amino acid composition but also in terms of their length. No examples of deletions or insertions were found. Therefore, it can be assumed that all analyzed sequences of plant ureohydrolases, and sequences in the *InterPro* superfamily of ureohydrolases (IPR006035) are in fact the same ARGAHs. They should present not only very similar structure to *At*ARGAH1 and *Mt*ARGAH but also they should share the same substrate binding mode and the same substrate specificity, as previously suggested (Patel et al., 2017).

### Comparison With Other Ureohydrolases

Examples of structures of other ureohydrolases with ornithine are found in the Protein Data Bank (PDB), e.g., for bacterial arginase from *Bacillus caldovelox* (PDB ID: 4CEV) (Bewley et al., 1999) or human arginase, *Hs*ARG1 (PDB ID: 3GMZ) (Ilies et al., 2011). Although bacterial arginases are usually hexamers and eukaryotic are trimers (Ahn et al., 2004), they bind ornithine in a very similar fashion. However, the binding mode is very different to that observed in plant ARGAHs (**Figure 6B**). The carboxyl group of ornithine in plant ARGAHs occupies the position of the amino group of ornithine in mammalian and bacterial arginases. This significant difference in the ligand binding mode is a consequence of the difference of loop *L*4 architecture. In *At*ARGAH1 there is Phe194 that fills this part of the pocket (**Figure 6B**). Additionally, bacterial and mammalian arginases basically lack *L*2* coil region. In *Hs*ARG1 this fragment is 9-residue shorter in comparison to *L*2* of *At*ARGAH1 and it forms a helix, which does not interact with a substrate. Therefore, the active sites of mammalian and bacterial arginases are independent in each subunit. Looking only at this comparison, it is no surprise that plant ARGAHs are not phylogenetically closer related to arginases from other domains of life than to bacterial agmatinases (Chen et al., 2004).

There are 36 structures of different proteins in the PDB that present the arginase topology annotated by CATH (Sillitoe et al., 2015); 25 of them are actual or putative ureohydrolases. Unfortunately, there is no structural data about ligand binding in other proteins than, already mentioned, arginases. It is worth noting that the structure of agmatinase from *Deinococcus radiodurans* (PDB ID: 1WOG) (Ahn et al., 2004) is annotated as the complex with product analogue, 1,6-diaminohexane, but a close look at the available electron density maps puts the interpretation of this ligand as highly doubtful. Therefore, information about this ligand was omitted in further analysis. Search across the PDB identified only a few proteins that might engage a neighboring subunit for the purpose of ligand binding, similarly to plant ARGAH. These are: procalvaminate amidinohydrolase from *Streptomyces clavuligerus* (PDB ID: 1GQ6), guanidinobutyrase from *Pseudomonas aeruginosa* (PDB ID: 3NIO) (Lee et al., 2011), 3-guanidinopropionase from *P. aeruginosa* (PDB ID: 3NIP) (Lee et al., 2011), putative agmatinase from *Clostridioides difficile* (*Cd*AGM, PDB ID: 3LHL), agmatinase from *Thermoplasma volcanium* (*Tv*AGM, PDB ID: 3PZL), and agmatinase from *Burkholderia thailandensis* (*Bt*AGM, PDB ID: 4DZ4).

**Figure 7:**
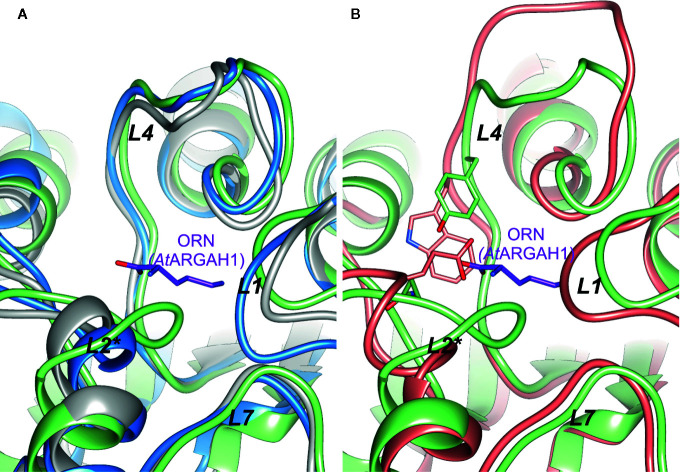
Comparison of plant AGAH with agmatinases. **(A)** Superposition of *At*ARGAH1 (light green) with agmatinase from *Clostridioides difficile* (grey, PDB ID: 3LHL), agmatinase from *Thermoplasma volcanium* (blue, PDB ID: 3PZL); **(B)** superposition of *At*ARGAH1 (light green) with agmatinase from *Burkholderia thailandensis* (salmon, PDB ID: 4DZ4); ornithine bound in *At*ARGAH1 is shown as violet sticks; positions of Tyr191, Asn99 (both *At*ARGAH1), and corresponding to them Trp165 and Glu78 (both residues from *Burkholderia thailandensis* agmatinase) are shown as sticks representation.

The active site vicinity of *Cd*AGM and *Tv*AGM is very similar to plant ARGAHs (especially the conformation of *L*4, **Figure 7A**), but none of the mentioned proteins share the same ligand-binding residues that are conserved in plant ARGAHs (Ser97-Thr98-Asn99 motif in *L*2* or Tyr191 in *L*4 of *At*ARGAH1). Therefore, they would most likely differ considerably from plant ARGAHs in terms of the ligand binding mode. This is also true for *Bt*AGM, which significantly differs from plant ARGAHs in *L*4. It has Trp165 in position corresponding to Tyr191 in *At*ARGAH1, and Glu78 instead of Asn99 (**Figure 7B**). Glu78 of *Bt*AGM in the region corresponding to *L*2* of *At*ARGAH1 would be ideal binding counterpart for terminal amine of agmatine. These proteins do not seem to have residues that would easily interact with carboxyl group of the substrate; thus, they would be specific strictly to agmatine as a substrate. Therefore, the conserved features of plant ARGAHs put them as truly unique ureohydrolases.

## Conclusions

The presented crystal structures of *At*ARGAH1 and *Mt*ARGAH revealed the ligand binding mode in these hexameric enzymes. The conformation of all loop regions around the active site of both proteins is very similar. Therefore, it is not surprising that interactions with the reaction product inside the active site are nearly identical for both ARGAHs. Both enzymes engage the loop region *L*2* from the neighboring subunit to stabilize the ligand inside the active site. Combining these results with highly conserved features of plant ARGAHs, it is likely that all ARGAHs in the plant kingdom recognize their ligands in a similar fashion. Although the mechanism of guanidine moiety hydrolysis is universal among ureohydrolases of different domains of life, the characteristic ligand binding mode distinguishes plant ARGAHs from other eukaryotic and prokaryotic arginases and agmatinases. These features seem to be an accommodation for the dual arginase/agmatinase activity of plant ARGAHs.

## Data Availability Statement

The coordinates and structure factors of the related structures were deposited in the Protein Data Bank (PDB): 6VSS (*Mt*ARGAH), 6VST (*Mt*ARGAH-ORN), 6VSU (*At*ARGAH1-ORN).

## Author Contributions

The author confirms being the sole contributor of this work and has approved it for publication.

## Funding

This project was supported in part by the Intramural Research Program of the National Cancer Institute, Center for Cancer Research.

## Conflict of Interest

The author declares that the research was conducted in the absence of any commercial or financial relationships that could be construed as a potential conflict of interest.
