# Supplementary material for: The Neighboring Subunit Is Engaged to Stabilize the Substrate in the Active Site of Plant Arginases
Source: Front Plant Sci. 2020 Jul 10;11:987. doi: 10.3389/fpls.2020.00987 (PMC7370999; doi:10.3389/fpls.2020.00987)
Supplement: Supplementary file 1 [file DataSheet_1.pdf]

Supplementary Information

The neighboring subunit is engaged to stabilize the substrate in the active site of plant arginases

Bartosz Sekula

Synchrotron Radiation Research Section, Macromolecular Crystallography Laboratory, National Cancer Institute, Argonne, IL, USA

The list of GenBank accession numbers of the final set of unique sequences of plant ureohydrolases found with BLAST search

>AAK07744.1
>AAK15006.1
>AAM64858.1
>ABK23295.1
>ABK26575.1
>ABR13881.1
>ACF85494.1
>ACJ84926.1
>ACU21474.1
>AFK40675.1
>AGR45902.1
>AIC73637.1
>AIC73638.1
>AIC73639.1
>AIZ68130.1
>AZL41258.1
>AZL93838.1
>B8AU84.1
>BAI22841.1
>BAJ96580.1
>CAA0394185.1
>CBI38896.3
>CDO97707.1
>EMS66394.1
>EPS67114.1
>GAV78653.1
>GBG90544.1
>GER31230.1
>KAA8520500.1
>KAB2595447.1
>KAB2595466.1
>KAD4982556.1
>KAD4982557.1
>KAE8715260.1
>KAE8791917.1
>KAE9460000.1
>KAE9603213.1
>KFK32106.1
>KHG24760.1
>KJB59654.1
>KMZ57542.1
>NP_001140646.1
>NP_001233851.1
>NP_001234578.1
>NP_001237121.2
>NP_192626.1
>NP_192629.1
>O49046.1
>OEL25084.1
>OMO79000.1
>OTF92261.1
>OVA19534.1
>PHT59131.1
>PIA50072.1
>PKA46562.1
>PLY89415.1
>PON34630.1
>PON37702.1
>PSS21573.1
>PTQ34191.1
>PUZ46057.1
>PUZ46059.1
>PWA46778.1
>PWA83066.1
>QCE02944.1
>RAL54061.1
>RCW19469.1
>RLM73259.1
>SPT15713.1
>TEY85548.1
>THU73124.1
>TKR75244.1
>TKS14668.1
>TMW93909.1
>TMW93910.1
>TQE01157.1
>TVU06875.1
>TXG62678.1
>TYI28885.1
>VAH24838.1
>VAH24839.1
>VDC91221.1
>VVA38968.1
>VVB08601.1
>VYS62047.1
>XP_002301277.2
>XP_002320051.1
>XP_002446022.1
>XP_002511686.1
>XP_002960232.1
>XP_002967483.1
>XP_003549862.1
>XP_003580734.1
>XP_003605169.2
>XP_004145005.1
>XP_004306935.1
>XP_004506527.1
>XP_004974987.1
>XP_006288104.1
>XP_006288117.1
>XP_006347880.1
>XP_006347881.1
>XP_006397249.1
>XP_006445281.1
>XP_006573429.1
>XP_006652085.1
>XP_006838611.1
>XP_007019836.1
>XP_007134852.1
>XP_007218271.2
>XP_008232616.1
>XP_008372715.1
>XP_008460103.1
>XP_008784644.1
>XP_009134113.1
>XP_009396988.1
>XP_009400802.1
>XP_009628685.1
>XP_010056331.1
>XP_010098932.1
>XP_010264188.1
>XP_010421796.1
>XP_010421799.1
>XP_010436235.1
>XP_010436279.1
>XP_010455297.1
>XP_010541873.1
>XP_010557557.1
>XP_010661662.1
>XP_010692213.1
>XP_010919046.1
>XP_011017479.1
>XP_011034056.1
>XP_011090497.1
>XP_012083598.1
>XP_012446520.1
>XP_012843587.1
>XP_013622543.1
>XP_013735088.1
>XP_014516328.1
>XP_015079755.1
>XP_015636156.1
>XP_015869964.1
>XP_015950638.1
>XP_016184161.1
>XP_016575182.1
>XP_016698236.1
>XP_017236069.1
>XP_017442602.1
>XP_017980777.1
>XP_018473157.1
>XP_018505555.1
>XP_018729674.1
>XP_019189201.1
>XP_019189207.1
>XP_019248444.1
>XP_019447164.1
>XP_020085035.1
>XP_020194637.1
>XP_020212207.1
>XP_020261722.1
>XP_020411945.1
>XP_020549031.1
>XP_020549034.1
>XP_020570867.1
>XP_020676907.1
>XP_020877484.1
>XP_020878486.1
>XP_021287778.1
>XP_021611851.1
>XP_021668530.1
>XP_021677880.1
>XP_021748011.1
>XP_021756289.1
>XP_021831197.1
>XP_021864267.1
>XP_021864269.1
>XP_021889543.1
>XP_021889544.1
>XP_021889545.1
>XP_021889546.1
>XP_022015661.1
>XP_022015662.1
>XP_022016080.1
>XP_022155763.1
>XP_022718347.1
>XP_022733756.1
>XP_022886366.1
>XP_022959268.1
>XP_022964547.1
>XP_023000235.1
>XP_023514911.1
>XP_023548510.1
>XP_023758557.1
>XP_023876199.1
>XP_024170839.1
>XP_024170851.1
>XP_024376768.1
>XP_024636707.1
>XP_024925040.1
>XP_024987441.1
>XP_024987442.1
>XP_025657342.1
>XP_025825031.1
>XP_026380488.1
>XP_026410702.1
>XP_027160661.1
>XP_027160662.1
>XP_027335678.1
>XP_027771541.1
>XP_027908392.1
>XP_028106459.1
>XP_028115883.1
>XP_028801201.1
>XP_028801202.1
>XP_028961471.1
>XP_030471018.1
>XP_030471019.1
>XP_030471020.1
>XP_030489906.1
>XP_030551990.1
>XP_030931986.1
>XP_031113935.1
>XP_031113936.1
>XP_031393732.1
>XP_031503571.1
